# What drives community adherence to indoor residual spraying (IRS) against malaria in Manhiça district, rural Mozambique: a qualitative study

**DOI:** 10.1186/1475-2875-10-344

**Published:** 2011-11-23

**Authors:** Khátia Munguambe, Robert Pool, Catherine Montgomery, Carlos Bavo, Ariel Nhacolo, Lina Fiosse, Charfudin Sacoor, Delino Nhalungo, Samuel Mabunda, Eusébio Macete, Pedro Alonso

**Affiliations:** 1 Centro de Investigação em Saúde de Manhiça, Rua 12, CP 1929, Manhiça, Mozambique; 2 Centro de Reserca en Salud Internacional de Barcelona, Hospital Clinic/Universitat de Barcelona, Roselló 132, 08036 Barcelona, Spain; 3 Centre for Global Health and Inequality, University of Amsterdam, OZ Achterburgwal 185, 1012 DK, Amsterdam, the Netherlands; 4 Institute for Science, Innovation and Society, Saïd Business School and Oxford Martin School, University of Oxford, Oxford OX1 1HP, UK; 5 Centro de Estudos Africanos, Universidade Eduardo Mondlane, Av. J. Nyerere 3453 - Campus Universitário, Maputo, Mozambique; 6 Faculdade de Medicina, Universidade Eduardo Mondlane, Av. Salvador Allende 702, CP 257, Maputo, Mozambique; 7 National Directorate for Health, Ministry of Health, Av. Eduardo Mondlane/Salvador Allende, CP 264, Maputo, Mozambique

## Abstract

**Background:**

Malaria control remains a challenge in sub-Saharan Africa. In 2006, the World Health Organization (WHO) reinforced the recommendation of indoor residual spraying (IRS) with dichlorodiphenyltrichloroethane (DDT) to reduce malaria transmission. The National Malaria Control Programme has been reporting high coverage rates of IRS in Mozambique. It is important to establish to what extent these rates are a reflection of community acceptability, and to explore the factors associated with adherence, in order to recommend suitable approaches for interventions of this nature.

**Objective:**

To understand the implementation process, reception and acceptability of the IRS program in Manhiça district, Southern Mozambique.

**Methods:**

Qualitative data was collected through in-depth interviews, participant observation of IRS activities, informal interviews, and focus group discussions. Study participants comprised householders, community leaders, health care providers, sprayers, and community members. Qualitative data analysis was based on grounded theory. Secondary data from the Manhiça Demographic Surveillance System was used to complement the qualitative data.

**Results:**

IRS was well received in most neighbourhoods. The overall coverage rates varied between 29% and 41% throughout the study period. The factors related to adherence to IRS were: immediate impact on insects in general, trust and obedience in the health authority, community leaders' influence, and acquaintance with the sprayers. Fighting malaria was not an important motivation for IRS adherence. There was a perception of limited efficacy of IRS against mosquitoes, but this did not affect adherence. Non-adherence to the intervention was mainly due to the unavailability of key householders, disagreement with the procedures, and the perception that spraying increased the burden of insects. Most respondents strongly favoured bed nets over IRS.

**Conclusion:**

The study suggests that the contribution of IRS to malaria and mosquito control is not entirely perceived by the beneficiaries, and that other as cost effective interventions such as insecticide-treated nets are favoured over IRS. Adherence to IRS was found to be influenced by socio-political factors. There is a need to redefine the community sensitization approaches in order to make IRS a genuinely participative, acceptable, and sustainable programme.

## Background

Malaria is an important cause of mortality in Africa, where 709, 000 deaths attributable to this infection occurred in 2009 [1]. In Mozambique malaria represents 44% of the external consultations in the health facilities, 60% of hospital admissions among children [2], and close to 26% of hospital deaths [3]. Despite the recent decline in the burden of malaria linked to the recent deployment and scale-up of effective control tools [4], malaria control in sub-Saharan Africa remains a public health challenge [5,6]. The World Health Organization (WHO) recommends indoor residual spraying (IRS), including with dichlorodiphenyltrichloroethane (DDT), as a malaria vector control measure [7].

In over 60 years of malaria control programmes in Southern Africa, Mozambique experienced a number of shifts in strategy and focus, reflecting in part the different historical eras that the country went through.

IRS with DDT was introduced in the 1940s in selected areas of Southern Mozambique [5,8], because malaria was a threat to the Portuguese settlers [9]. With the selection of the southern region of Mozambique for the implementation of WHO's pre-eradication programme, IRS with DDT was intensified in the early 1960's [10], but it was scaled down in the late 1960s, as a reflection of the abandonment of the eradication commitments observed worldwide [5,9]. Despite the civil war, which started in the late 1970s, and its negative impact on the national health system, IRS continued to be implemented through the 1970s [10] and the campaigns did not halt until the 1980s. IRS was reintroduced in the early 1990s in selected areas [5].

In 1999, the Lubombo Spatial Development Initiative (LSDI), a three-country malaria control programme led by South Africa, was established to safeguard the tourism and agriculture economic potential in Kwazulu Natal, which borders southern Mozambique and Swaziland [8,11]. Since 2005, the implementation of IRS has been LSDI's major intervention, which is financially supported by the Global Fund against AIDS, Tuberculosis and Malaria, the South African Government, and the private sector [5]. This malaria control approach may be more suitable to the South African context where the dimension of the problem is not comparable to Mozambique's, because while malaria is endemic in Mozambique, its transmission in South Africa is very low with hot spots of medium transmission, and only 4% of the population at high risk [1]. Further and differently from Mozambique, malaria in South Africa is viewed as an economic rather than a major public health problem [12].

Despite initial government reservations towards DDT, it was reintroduced in Mozambique in 2005, and since then IRS programmes using DDT have scaled up countrywide. This choice in strategy was further supported by WHO's recent position statement recommending IRS, including with DDT, as one of the primary interventions to be scaled up in endemic areas [7].

It is reported that the campaigns have been reaching the required coverage countrywide [13,14], raising the assumption of strong community support [8] resulting from a recognition of the intervention's potential benefits, as suggested by a number of health behaviour theories.

The Health Belief Model, on which many health interventions stand, holds that adherence to a health intervention is a result of a positive expectation that by taking a recommended action a negative health condition will be avoided [15]. However, this argument can be challenged when it comes to interventions such as IRS implemented in contexts such as rural Mozambique, where anecdotal evidence has suggested an apparent acceptance of IRS despite the local sense that IRS is an inefficacious tool [16]. Similarly, avoidance of malaria was not found to be a key motivation for the high rates of IRS acceptability found in Mexico [17]. This raises a number of questions that are important from a health promotion perspective and consequently deserve further study. In particular, it is of interest to understand the reasons why large sections of a community can accept a health intervention that they nonetheless perceive as not efficacious. It is also important to continue to identify factors determining adherence to the intervention, overriding the factors that can trigger rejection.

Most studies on people's practices related to malaria control in the context of ongoing interventions in Africa have focused on acceptability of interventions involving drugs and insecticide-treated nets (ITNs) [18,19]. Published information on community's reception and acceptance of IRS is limited to reports on coverage levels [18,20]. The evidence on factors underlying community acceptance of IRS is scarce, with only a few studies that identified reasons for acceptance or refusal and motives of satisfaction [17]. Montgomery and colleagues describe in detail the political and historical influences on acceptability for IRS in Southern Mozambique [21]. The informal processes and practices, the local knowledge embedded in these practices, and the influencing socio-cultural and environmental contexts which are essential to the success of IRS as a community-based intervention are yet to be explored.

The present study, which builds on Montgomery's analysis [21], aimed to gain an in-depth understanding of the local dynamics of the implementation and community acceptability of an ongoing indoor-residual house spraying intervention in a rural community of southern Mozambique.

## Methods

### Study site and population

The study took place in the District of Manhiça, which is 80 km North of Maputo city. The District covers an area of 2,360 Km^2 ^and its population in 2007 was 157,642 inhabitants [22].

The district is divided into six administrative posts, each of which is subdivided into localities (*localidades*), villages (*aldeias*) and neighbourhoods (*bairros*). The leaders at these levels (village or locality presidents, and neighbourhood secretaries) perform the local political and administrative functions and report to the District level.

The majority of the district's population live in the Administrative Post of Manhiça-Sede and belong to the Changana ethnic group. They are mostly subsistence farmers and employees of the Maragra and Xinavane sugar estates. A significant number of people is engaged in informal trade. Manhiça is also a source of migrant labour to South Africa, Swaziland and Maputo City. Illiteracy rate among adults was 78% in 2008, being more prevalent among women [23].

**Figure 1:**
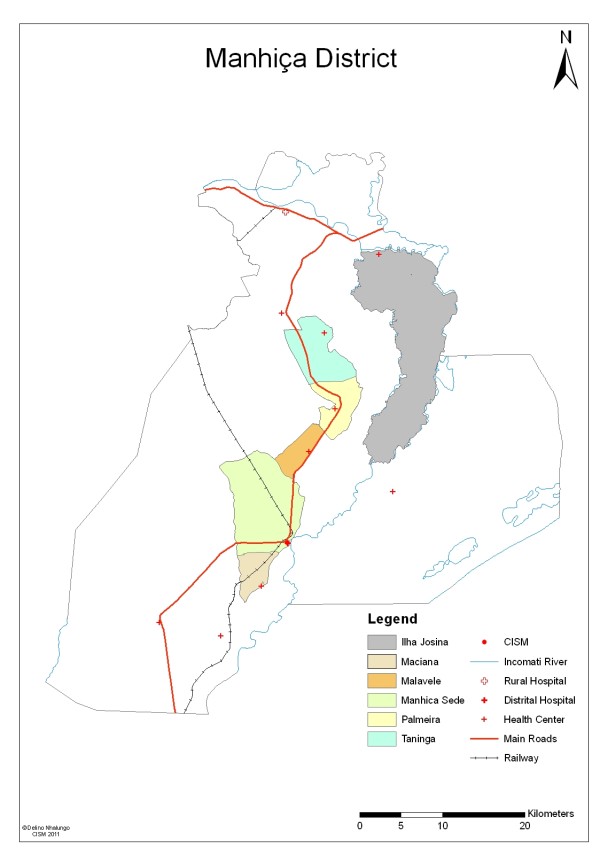
**Map of Manhiça District**. The map of the district of Manhiça, highlighting the six residential areas which belong to the Manhiça Demographic Surveillance Area and were involved in the study.

A continuous Demographic Surveillance System (DSS), that has been described elsewhere [24], has been running since 1996, currently covering a population of around 86,000 inhabitants, within an area of around 500 Km^2 ^(Figure 1). Within this area, households are are grouped into six geographical areas: Manhiça-Sede (main town and surroundings), Maciana (10 Km south of Manhiça-Sede), Malavele (13 Km north-west of Manhiça-Sede), Palmeira (18 Km north of Manhiça-Sede), Taninga (37 Km north-west of Manhiça-Sede), and Ilha Josina (58 Km north of Manhiça-Sede).

Malaria transmission is perennial, with seasonal peaks during and after the rainy season (from November to March). *Anopheles funestus *is the main malaria vector, and in 1998 the estimated entomologic inoculation rate was 15 infective bites per person per year [25]. In Manhiça, malaria accounts for almost 40% of the outpatient visits and 69% of hospital admissions in children aged two years or older [26,27]. IRS activities began in 2005, and since then at least one campaign per year has taken place.

### Data collection

The main approach used in the study was Ethnography, which allows the researcher to see the world through the study community's eyes and understand in detail how beliefs and practices are embedded in local norms and cultures [28]. In order to achieve this, part of the study fieldworkers lived in the study neighbourhoods, where they participated in the daily life of the communities.

As shown in Table 1, a combination of methods namely in-depth interviews (IDIs), Focus Group Discussions (FGDs), participant observations, and informal conversations, were used to collect qualitative data on community's expectations and experiences regarding IRS, perception of effectiveness, reasons for acceptance or refusal to collaborate with the intervention, and satisfaction.

**Table 1:** Qualitative study methods.

	Target groups and sample size	
Data Collection tool		
	IRS-1	IRS-2
In-depth interviews (IDI)	12 community leaders	10 community leaders
	50 householders	32 Householders
	11 health care providers	16 sprayers

Focus group discussions	21 FGD with householders (8 pre-spray and 13 post-spray)	1 FGD with sprayers
(FGD)	2 FGD with health care providers	

	2 FGD with sprayers	
Informal conversations	-	15 community members 5 sprayers

Participant observation	-	17 households

Qualitative data collection tools and target groups involved at each study stage

These data were collected in two stages. The first stage (IRS-1) took place at the earliest stages of IRS implementation from January to April 2006. The second stage (IRS-2) was conducted from January 2007 to November 2008.

Quantitative data on IRS coverage was obtained through household surveys routinely conducted as part of the DSS. The surveys were conducted at six-monthly intervals. In each household, a questionnaire was administered to the head of household, or an adult over 18 years of age, who provided information on whether the household had been sprayed within the previous 6 months and, when possible, was asked to show the household spraying certificate. Households that were not sprayed were asked about the reasons for not spraying.

### Sampling and recruitment strategies

For the qualitative data, the Grounded Theory approach was followed [29], whereby data was collected until theoretical saturation was reached, i.e. until no additional data were being generated that could add new interpretive categories or further insights to existing ones.

Recruitment for in-depth interviews with householders in IRS-1 was by convenience: householders were identified by local fieldworkers or community leaders, and snowballing was then used to recruit further participants until saturation. In IRS-2 at least five households per residential area (shown in Figure 1) were randomly selected using the DSS database. Further households were randomly enrolled until saturation. In both stages a number of non-adherent households were included in order to minimize bias introduced by self-selection of the most adherent households.

Recruitment for the FGDs and informal conversations was by convenience: for the informal conversations, respondents were recruited from places such as markets, water sources, and the streets whenever it was found appropriate and convenient to develop a discussion around IRS. For the FGDs, recruitment took place in the communities or the health care facilities in collaboration with community representatives or health care providers.

In total, 131 in-depth interviews were conducted with community leaders, householders, health care providers, and sprayers, 20 informal conversations with community members, 26 focus group discussions with community members, health care providers and sprayers, and participant observation of sprayers' activities in 17 households (Table 1).

The DSS surveys included every household registered in the DSS, as shown in Table 2.

**Table 2:** IRS coverage throughout the study period.

Round (period, year)	Percentage of sprayed HH (range)	Total number of contacted HH
**Jan-Jun 2006**	29 (25 - 39)	18420
**Jul-Dec 2006**	34 (28 - 45)	20522
**Jan-Jun 2007**	41 (30 - 56)	25038
**Jul-Dec 2007**	40 (29 - 55)	24562
**Jan-Jun 2008**	41 (30 - 57)	24135
**Jul-Dec 2008**	39 (29 - 56)	24308

Households within the study area reporting IRS carried out within the previous 6 months, according to the DSS database

### Data management and analysis

Voice recorded in-depth interviews and focus group discussions were transcribed *in verbatim *and field notes from observations and informal interviews were typed. Transcriptions and field notes were imported into *Nvivo 2.0 *[30], a computer programme that facilitates the management and coding of large sets of qualitative data.

Qualitative analysis was done through grounded theory, which allows theoretical generalizations to emerge gradually from the data, as opposed to pre-determined hypotheses [29]. Passages of text were initially categorised into common general themes. With further reading of the texts, additional occurrences of the categories were identified and labelled accordingly. Secondarily, similarly coded text was retrieved to allow branching out of sub-themes and relationships between them. When relationships between two or more nodes began to surface, the questioning and comparison method was employed. This was done through systematic searches for passages containing likely key words representative of the suggested relationships, in order to verify the degree of recurrence of such relationships throughout the transcribed material.

Data from DSS surveys on IRS coverage corresponding to the period from January 2006 to December 2008 was considered for quantitative analysis. Descriptive statistics on sprayed households was performed using STATA 10.0 [31]. One-way contingency tables were generated to calculate the proportion of households which had been sprayed within the previous six months (considered as adherents for the purpose of this study), and to calculate the frequency distributions of the responses regarding reasons for non-adherence.

### Ethical issues

Ethical clearance was obtained from the Science and Ethics Committee of the London School of Hygiene and Tropical Medicine in the UK, the National Health Bioethics Committee in Mozambique, and the Hospital Clinic of Barcelona Ethics Review Committee in Spain, followed by administrative approval by the Mozambican Ministry of Health.

Written informed consent was sought from all participants involved in in-depth interviews, and verbal informed consent was obtained from participants of informal conversations, FGDs, and participant observation. Participant's consent was recorded on all occasions when digital tape recorders were used. All participants were only identifiable through unique identification numbers to guarantee confidentiality.

## Results

### Local dynamics of IRS implementation

Throughout the study period one spraying round per year took place in Manhiça. The rounds were referred to as *campanhas de pulverização *(spraying campaigns), which started a few months before the rainy season and were planned to last 90 days.

IRS was performed by spraying teams, the so-called *brigadas *(brigades), which comprised up to 20 young male and female individuals. Most of the sprayers had secondary level of education, and in addition had received a 10-day training course on IRS. Some sprayers compared the teaching methods employed in the training course with military training approaches, in the sense that the sessions were physically hard, some of the information was conveyed in the form of commands, and more attention was paid on their physical and technical preparedness rather than the theoretical aspects of IRS and malaria control.

*It [the can] had 10 litres. We would take that water on our backs...we would rest for 10 minutes after each hour, I think, and we would take that water everywhere...even to the bathroom. This was the training for 10 days, from morning until 16:00 hours*. - IRS2, IDI, male sprayer

Once in the field, the entire District was covered by one brigade, under the coordination of a field supervisor who, among other roles, was responsible for community sensitization activities.

The IDIs and FGDs with community members revealed that the majority of respondents had some level of awareness of the presence of the brigades in their neighbourhoods during the first days of the campaigns. Many respondents said to have heard of the campaigns through the radio. Additionally, sprayers reported that up to one week before the kick off of the campaign, brigade supervisors gave notifications to the *secretários dos bairros*, which in turn organized community meetings to sensitize them to collaborate with the spraying brigades.

After that, the brigade would start spraying the houses within the notified areas. Some secretaries would chose to conduct door to door mobilization on the spraying days, most with the aid of megaphones, and others opted to have their own house sprayed to set an example. According to the sprayers, not all *secretários *were able to entirely reach their target audience through these approaches, since they came across many households that were taken by surprise on the spraying day.

Sprayers regretted that there were some, although very few, *secretários *who were not willing to collaborate and because of that they found themselves improvising door-to-door mobilization sessions on many occasions.

*We talk to the head of household and we say: we did not come to your house to harm you. We are here to protect you so that you have good health. Malaria is a disease that kills many people, and we have a method that prevents it...since the start of the spraying the disease has reduced until now*. - IRS2, FGD, male and female sprayers

It was found through the participant observations that the key message conveyed to the householders was that the brigades' mission was to combat the malaria-causing mosquito. It was noted that in the case of refusal, the message was repeated with the intention to change the householder's mind, but the content of the message was hardly modified in order to meet the refusing householder's concerns.

Since the information conveyed during the community mobilization was unilateral, the only occasion for householders to clarify any queries regarding IRS was the day of the spraying. However, only one out of 17 observed households raised questions to the sprayers. Sprayers reported that in general community members wanted to know the mechanisms through which they select the malaria-causing mosquito and recognised their limitations in answering such question. Below is one sprayer's attempted explanation:

*This was studied by the experts, it was seen that the type of medicine [spraying product] that we are using...the measurement of water that we use for the dilution is exactly proportional to kill the anopheles and no other mosquito*. - IRS2, FGD, male and female sprayers

At the households, once consent was obtained, sprayers asked householders to keep the walls free of any object or furniture to facilitate the spraying. Householders received instructions to maintain the doors and windows closed and to keep eating utensils and foodstuffs outside the house for at least two hours after the spraying. After that period doors and windows should be open for 1 hour while sweeping the interior of the house and burying the resultant residues to prevent children and domestic animals from ingesting dead, poisoned, insects.

Sprayers were generally viewed as respectful because they approached the households and asked for consent using locally accepted manners. They presented themselves as "The Health", which in turn was associated with "The Government" to add legitimacy to their work. The sprayers were also members of the communities, resulting in an unclear line between the intervention providers and the receiving communities, and this has acted in favour of rapport building between the two parties.

*Even in one of the houses that I sprayed today, the owner ended up letting me know that he was respecting me because he knew me, If he did not know me he would never have allowed me [to spray]. - *IRS2, FGD, male and female sprayers

Sprayers' liaison with the communities was seen on two further levels. First, there were instances when sprayers had to spray their own houses. From that emerged the perception that they could spray as they wished.

*My house was last sprayed last month. I don't know how many times it was sprayed last year because since my husband is a sprayer, he does it very often...all I know is that it was more than 4 times...I don't know if he will be able to spray again because now he works far from here and they no longer allow to bring the product home*. - IRS2, informal conversation, adherent female respondent

Likewise, there was the perception that people who were acquainted with sprayers were privileged from having their households sprayed with good quality products.

*Look, you talk like that maybe because you have a sprayer friend who offers you the good quality medicine [spraying product] and you always spray when you need to spray, because all the [other] people close their doors but the medicine doesn't last for long*. - IRS2, informal conversation, two male traders at the market

While there were households who were passive receivers and not in control of their house's fate regarding the intervention, a few householders were more proactive in demanding the service by warning sprayers not to skip their houses, or by literally stopping passing by sprayers and demanding them to spray their houses there and then.

### Community reception of IRS

FGDs conducted in early 2006 in the areas where spraying had never occurred revealed positive expectations, in the sense that IRS would reduce the nuisance created by mosquitoes and other insects. Reduction of malaria was a few times mentioned among many other health problems perceived to be caused by mosquitoes. These expectations were particularly strong in Ilha Josina, Palmeira, and Taninga.

Another group of neighbourhoods, namely Malavela, and two sections of Manhiça-Sede, were not convinced about the advantages of this intervention, especially because of the already circulating rumours, at the earliest stages of IRS implementation, which pointed to an increment of insects due to IRS.

To most participants, even in the areas with high expectations towards IRS, there was awareness of possible limitations of IRS such as durability of the effect and the risk of being bitten by mosquitoes in unsprayed areas.

Despite the perceived limitations, people were willing to experiment IRS and experience the alleged disadvantages for themselves.

Only one neighbourhood reported having few mosquitoes in the area, although recognizing the burden of malaria and many other diseases. In their view such health problems were caused by *moya *(local term meaning "air", "wind", or "season", in this case as a vehicle of disease transmission), therefore IRS was not viewed as an appropriate solution.

*They have to go to the places where there are mosquitoes. We do not want them to spray our houses because we do not have mosquitoes here*. - IRS1, pre-spraying FGD, male and female community members

DSS data gathered within the period of the study indicated overall coverage rates between 29% and 41% (Table 2). Still according the DSS, there were marked variations in coverage levels across the 6 geographical areas, with the Maciana and Malavele areas registering invariably lower coverage rates, while Ilha Josina and Palmeira consistently registered the highest rates over the study period (Table 3).

**Table 3:** IRS coverage per residential area throughout the study period.

	Year	2006		2007		2008	
	
	Round	Jan-Jun	Jul-Dec	Jan-Jun	Jul-Dec	Jan-Jun	Jul-Dec
Residential area							
Maciana		13.9	5.8	8.4	7.5	10.7	6.1
Manhica-Sede		36.9	37.6	38.3	41.5	54.7	52.6
Malavele		3.2	3.6	27.3	1.6	0.1	0.3
Palmeira		27.2	49.9	66.4	60.8	40.7	39.5
Ilha Josina		35.1	58.2	73.8	79.1	76.2	77.4
Taninga		58.9	54.5	49.7	53.0	66.0	66.3

Percentage of Households by residential area reporting to have been sprayed within the previous 6 months, according to the DSS database

In terms of perceived coverage rates, the sprayers considered that there were more adherent households in comparison to non-adherent. They also reported that the coverage rates did improve with time.

There were neighbourhoods which were particularly known by the sprayers as having high numbers of non-adherents. These neighbourhoods belonged to three sectors within the Manhiça-Sede area. FGDs held within this area confirmed this perception among its own inhabitants. They were not happy with the exclusion of courtyards, public areas, and the bushes from the spraying. Rumours that spraying could increase the burden of other insects offered them more arguments for refusal.

*In fact in our neighbourhood they were not well received because the mosquitoes breed and live outdoors in the bush and they said they wanted to spray inside the house. This does not make sense*. - IRS1, post-spraying FGD, male and female community members

### Factors influencing adherence to the campaigns

The main factors influencing adherence to the campaigns are summarized in Table 4. IDIs held in the pre-spraying period revealed very few participants with a thorough understanding of the concept, purpose, and benefits of IRS and who were willing to accept spraying for those reasons. Fighting malaria was a rare expectation of IRS (mentioned by 2 out of the 27 respondents who voiced their expectations). On the other hand, there were many respondents who were willing to accept IRS, although they were unable to explain its purpose and were not aware of its health outcomes.

**Table 4:** Reported reasons for adhering and not adhering to IRS, according to IDIs and FGDs

Reasons for adherence to IRS	Reasons for non-adherence to IRS
• Immediate killing action on insects (mosquitoes, fleas, ticks, and cockroaches) • Disease avoidance in general • Fulfilment of Governmental Orientations - *the law of the hospital* • Fear of punishment from health authorities • Fulfilment of citizenship duties • Trust/acquaintance between householders and the sprayers • Community leaders' involvement and persuasion • Good references from previous experience with IRS	• Short-notice or no notification of IRS brigade's presence in the neighbourhood • Absence or time limitations of household decision makers to consent and facilitate the procedures • Perceived limited effectiveness on mosquito control • Perceived long-term increment of fleas, ticks, and cockroaches • Lack of understanding of/disagreement with spraying procedures • Non-involvement of credible community structures, other than political entities, in the sensitization activities (ex: churches, associations) • Community leaders' non-adherence to IRS

Once the campaigns started, householders revealed that their previous first-hand experience with IRS contributed to their collaboration in the subsequent rounds. There were others who, by having observed or experienced IRS in neighbouring households and other regions such as Maputo and South Africa, became interested in having their own houses sprayed. However, being aware of other people's perceived negative effects of IRS was not always a driver of refusal.

In many cases, householders justified their decision to adhere to IRS on their trust on the judgement made by third parties. "The Government", "The State", "The Ministry of Health", "The District Health Authorities", most commonly referred to as simply "The Health", and "The Hospital" were viewed as a single institution, which was responsible for IRS implementation. In general, people trusted "The Health" with the power and competence to make decisions regarding their health matters on their behalf. In their view, this was because the Government was in the best position to evaluate risks against benefits of undertaking any health intervention, and therefore would never do harm to "their" people.

*They said it is a good thing because it is about help from the Government. That idea is welcome, now those who say that hey something bad will happen, it is not possible, the Government cannot wish to kill us, it was the Government who said that when they arrive we must open the doors to them because they are protecting us. We want to live therefore we cannot refuse. - *IRS1, pre-spraying FGD, male and female community members

A number of householders expressed a sense of belonging to the intervention, describing themselves as "members of the spraying" or "collaborators of the programme". Similarly, many respondents proudly showed off the spraying certificate. In their view, it served additional purposes besides accountability, namely as a certificate of participation, as an identification mark for non-refuters, and as a sign of commitment with future campaigns or other governmental programmes.

In a few cases the discourse suggested an intention to fulfil a greater good. The fact that the intervention was taking place in the whole country was highlighted in the messages delivered by the radio and community leaders, giving community members reassurance and legitimacy to their choice to adhere the intervention as part of a greater cause. However, the tone in some leaders' discourses, using expressions such as "no-one must leave their houses" and "you must all accept the intervention" reveal power relationships, suggesting that the decision making process did not allow enough room for independent judgements. This was in agreement with what the sprayers considered as the main driver of adherence.

*The people accept the spraying because it has to do with a law *- IRS2, IDI, male sprayer

One final factor motivating adherence to IRS was status. The inhabitants of Ilha Josina viewed IRS as a symbol of modernity, acting as a replacement of traditional mosquito deterrence practices such as the use of smoke repellents.

### Factors influencing non-adherence to the campaigns

Reasons for non-adherence are summarized in Table 4. Additionally, the DSS database provided the frequency distribution of reported reasons for not having the house sprayed, as seen in Table 5.

**Table 5:** Reasons for non-adherence to IRS.

	Percentage of non-adherent HH					
**Year**	**2006**		**2007**		**2008**	

**Round**	**Jan-Jun**	**Jul-Dec**	**Jul-Dec**	**Jan-Jun**	**Jul-Dec**	**Jan-Jun**
**Reason for non-adherence**						

Deliberate refusal	2.5	2.2	2.8	2.9	3.7	4.2
Absence	16.6	17.0	22.4	18.4	20.9	16.6
Brigade did not show up	78.8	76.5	67.9	70.1	70.7	73.7
Unknown	2.1	4.3	6.8	8.6	4.7	5.4

Total of non-adherent HH	11246	11341	11.045	11.058	10.457	10.828

Reported reasons for not having the house sprayed within the previous 6 months among non-adherent households, according to the DSS database

Non-adherents comprised not only those who deliberately refused the intervention, but also those whose houses were not sprayed for reasons other than unwillingness to accept IRS (Tables 4 and 5). As a result, over half of the interviewed non-adherents felt that they were unfairly labelled as "those who refused" even though they were actually willing to adhere to IRS but specific circumstances did not allow them to do so.

According to the DSS data, the vast majority of non-adherents claimed that their house had not been sprayed because de brigade had not shown up (Table 5).

According to the IDIs and FGDs, the most common reason for not having the house sprayed was the surprise factor caused by short-notice or no-notification of the spraying schedule, resulting in unpreparedness to allow the sprayers into the houses on the scheduled day. Another circumstance was the unavailability of the head of household or another key householder who could give consent to or facilitate the spraying procedures. This was most common to householders who were employed or engaged in farming, vending or other activities away from the neighbourhood. These households were likely not to be sprayed because according to the sprayers' accounts their tight schedules did not allow further visits to the unsprayed houses, unless a recovery round was planned.

Interviews with the so-called "adherents" revealed that even among them there were households which were not entirely sprayed as a reflection of the organization of households into compounds (related households organized as separate units within the same courtyard). Many respondents refused that certain units of their households be sprayed due to fears of contamination of foodstuffs and of objects employed in traditional rituals. Therefore cellars, kitchens, and areas allocated to traditional ceremonies (especially in the traditional healers' households) were often not sprayed.

*They did not spray because I had just returned from the "machamba" (cultivation field) and when they arrived the only huts which were ready [to be sprayed] were the spirit houses and in those places the spraying is not allowed. I asked them to comeback the following day so I could have my sleeping hut ready and they never came back*. - IRS1, IDI, non-adherent female respondent

Of those who deliberately did not adhere to IRS, the majority attributed this to a perceived ineffectiveness of IRS, based on their own or other people's past experiences.

*On the first day I slept well but on the second one it was not easy because there were a lot of mosquitoes. I thought that it would take two to three months without being bitten by mosquitoes, but that it was going to evaporate on the same day I did not know it... even my wife has malaria now*. - IRS1, IDI, non-adherent male respondent

Part of those who refused IRS had been influenced by misunderstandings regarding the mechanisms of action of IRS. Since the community mobilization messages emphasized that the product was specific to malaria-causing mosquitoes, but did not explain that this was due to the residual properties of the insecticide which took advantage of the specific feeding and resting habits of malaria-causing mosquitoes, suspicions were raised among community members who kept questioning how the product was able to select only one among the different mosquito species and how one could ascertain that the surviving mosquitoes did not transmit malaria. Still related to these misunderstandings, there was questioning as to how sensible it was to spray during the day while mosquitoes bit in the evening.

*... I do not believe that it [IRS] works because the mosquitoes increased. Unless if they sprayed at night, maybe it would be the opposite because I see that they [mosquitoes] are coming from the bush and from the neighbours..*. - IRS1, IDI, non-adherent female respondent

Likewise, it was hard to understand why spraying did not take place outdoors. Facing this concern, a number of householders reported negotiating with the sprayers in order to have at least the toilets and bathrooms sprayed but the sprayers explained to them that this was unnecessary without specifying the reasons. As a result there were households that ended up refusing IRS altogether.

Participants suspected that the product was poisonous because they witnessed its killing action on insects and pests. Surprisingly, this was not a factor triggering non-adherence as the same people that considered the product toxic reported that they had accepted IRS based on their trust on the implementers.

*The medicine is dangerous. It kills cockroaches, rats, ticks, everything, but we have to be careful because after they spray there is a deadline of twenty-four hours... That is why we must shut the doors and when we open it everything is dead. Children cannot pick anything from the floor otherwise they die...next day we do not see any cockroach dying and I think the medicine is no longer dangerous for children or us. - *IRS1, post-spraying FGD, male and female community members

Finally, the sprayers themselves considered that non-adherence was highly influenced by the sometimes inconsistent and non-involvement of community leaders throughout the process of the campaign.

### Community's satisfaction towards IRS

Aspects related to malaria control did not feature at all as a measure of satisfaction of IRS. In fact, most participants were not convinced that IRS was reducing mosquitoes or malaria in their homes and neighbourhoods, and believed that malaria could never be controlled through this intervention.

*...Even the war killed some [people] but other people like us are still here, remaining, just like the mosquitoes they cannot die all of them, there will always be remnants*. - IRS1, post-spraying FGD, male and female community members

With regards to mosquito reduction, for most of the respondents the expectations were only partially met. There was some level of satisfaction because mosquitoes and other insects diminished straight after the spraying, but on the other hand there was disappointment at the duration of the effect of the product. With this regard, virtually all IDI and FGD participants were unanimous in considering that the product's effect was short living, which was said to last between one day and a couple months. Only one participant reported that in his house the effect improved gradually with time.

Although the sprayers' messages emphasized the product's killing action against Anopheles mosquitoes, this was not an important effectiveness indicator in the perspective of the householders. People evaluated effectiveness partly based on the reduction of mosquito bites and elimination of all kinds of mosquitoes. FGDs conducted after the first campaign revealed that people in all participating neighbourhoods were not satisfied with the impact on mosquito reduction but were satisfied with the mid-term control of other pests.

*We did rest indeed on that week of the spraying because we did not have mosquito problems, but now they reappeared in large numbers... one week after spraying there are even more mosquitoes! But cockroaches, fleas, ticks, we no longer have those complications, they all died, but mosquitoes no. - *IRS1, post-spraying FGD, male and female community members

With a few exceptions of householders who felt clearly angry with the results of the spraying, most community members were willing to comply with future campaigns despite their disappointment in the intervention's effectiveness, and did not show hard feelings towards the intervention and its organizers. They continued holding that IRS was a good tool to fight mosquitoes and other insects, but blamed the failures on the duration of effect on the aspects listed below:

• *The use of new, less efficacious products*

• *The use of expired or counterfeit products*

• *Over dilution of the product (deliberately or accidentally)*

• *Product being suitable for concrete walls rather than reeds*

• *Householders failing to follow sprayers' instructions*

• *Spraying in the wrong season*

• *Perception that the best place to kill mosquitoes is outdoors*

• *House location near the river*

Because of the perceived limitations of IRS, even the households that were sprayed continued to resort to alternative, locally-available mosquito avoidance methods.

*I sleep under the bed net, and my family is in the other hut over there. When I see that hey they are being bitten by mosquitoes, I get up and prepare "sule" [smoke repellent], at least they will be able to sleep and that is not because it kills [mosquitoes] but it manages to reduce [mosquitoes], that is why nobody can stop burning "sule" *- IRS1, IDI, adherent male respondent

Moreover, the discourse favouring bed nets over IRS was recurrent, both from those who were already using bed nets and continued doing so even after IRS, and those who longed for bed nets despite the implementation of IRS.

*Bed nets are a good thing and are in the first place, because you get in there with your son and no mosquito bites you. If one [mosquito] enters it is because something went wrong when you left the bed o go to the toilet...we cannot stop using [bed nets] because as soon as that drug [IRS product] disappears the mosquitoes will reappear*. - IRS1, post-spraying FGD, male and female community members

## Discussion

Anecdotal evidence from previous fieldwork in Manhiça suggested that IRS was generally acceptable despite low levels of perceived efficacy [16]. This study provided evidence to support this argument by further revealing a perception that the effectiveness of IRS was short-lived. In spite of this perceived limited effectiveness, the DSS data revealed segments of the study area with coverage rates reaching 79%, although the overall coverage rates did not go beyond 41% throughout the study period. These figures were low compared to the official records, which point to coverage rates of around 56% in Maputo province and 52% at National level between 2006 and 2007 [32]. These differences could be because while official reports are representative at national and provincial level, DSS data accounts to about 60% of the Manhiça District population.

Further, the DSS covers the most densely populated areas of the district, the majority of which inhabiting the vicinity of the Manhiça Town. This population has characteristics consistent with slightly more urbanized lifestyles, which might have influenced adherence, thus influencing the overall DSS coverage figures. To support this supposition, the DSS records indicated that Ilha Josina and Taninga, which are the two most remote, most rural areas of the DSS area, registered the highest IRS coverage rates.

Different methodologies used to estimate IRS coverage rates could also have accounted for the discrepancies found between DSS and NMCP data.

Despite the differences between the overall IRS coverage figures from the NMCP and the DSS, the latter was useful in pinpointing important segments of the population, which did not adhere to IRS.

By further analysing these data, it shows that deliberate refusal was the least frequent reason for non-adherence. This is in line with what the national survey indicated [32] and suggests that non-adherence was not necessarily a reflection of rejection of IRS. The qualitative data supported this further by unpicking the circumstances leading to non-adherence, most of them beyond the householders' control. These were mostly related to incompatibilities between the campaign's and the householders' schedules. Importantly, the perception that the brigade did not show up was the most frequent reason for non-adherence. This is another finding that should be interpreted with caution because as these data were based on householders' recall, it could be a reflection of householders' absenteeism.

Consistent with findings elsewhere [17], reducing the burden of insects, including mosquitoes, inside the houses was by far the most important expectation and an important factor influencing adherence. This was regardless of a perceived reduction in the burden of malaria, as evidenced by the general belief that malaria could not be eliminated.

Moreover, the data indicate that in general, the understanding of the ultimate goal of the intervention was not necessarily a factor driving willingness to participate and actual adherence to IRS, because on one hand there were many respondents who were willing to accept the intervention but were unable to specify the health benefits of the campaign and on the other hand there were a few respondents with a thorough understanding of IRS but unwilling to adhere the campaign. This mismatch between knowledge of malaria control and adherence, together with the already discussed mismatch between adherence and perceived limited effectiveness of IRS is consistent with results on the non-correlation between malaria knowledge and adherence with malaria control interventions in Africa and elsewhere [17,33].

The community members discourse supporting outdoor spraying point to a deficient understanding of the logic behind IRS. This is of note not only among the householders but also among the sprayers themselves, who did not entirely grasp the concept of IRS. As a result, an inefficient dialogue between the implementers and the beneficiaries of the intervention could jeopardize community participation. Fortuitously, this did not seem to be a major issue in Manhiça, possibly because culturally and historically this population relies more on group based rather than individual based judgements in order to make health related decisions, as identified by Montgomery and colleagues [21] and discussed further below.

Without questioning the role of education and cognitive skills in determining conscious informed decisions in favour of an individual's health gains, the finding regarding deficient understanding of IRS, in contrast with the observed levels of adherence to the intervention challenges the health belief model and many other behavioural theories [15] as to what depth does a community in a setting like Manhiça need to understand the effect of spraying on malaria in order to guarantee adherence. In contrary, the present study suggests that if there is adherence, regardless of the reasons, then there is potential for the spraying campaign to take place successfully. The same has been reported in other interventions, for example the expanded programme on immunization (EPI) in African contexts, in which mothers hardly know what specific diseases the programme targets but the fact that they adhere to the vaccination programme has made the intervention successful [19,34].

Political and social factors were important drivers of adherence in Manhiça and they seemed to override the factors that could drive non-adherence. In particular, this study revealed resonance with militant mobilization approaches experienced in Mozambique in the context of pre- and post independence war. This was evidenced by the tagging of key components of the intervention, such as "brigades", "campaign", "fighting the mosquito enemy", as well as parallelisms made between the effect of the war on people and of IRS on mosquitoes, to name but a few.

Many people adhered to the intervention in obedience to the "hospital law", which is a recurrent socially constructed concept, also described elsewhere [34,35] that comprises codes of conduct in the interaction between the population and the Health Authorities, whereby the "The Health" has the role of delivering the services to which the community is supposed to adhere. Although there was no clarity of what people thought could be the repercussions of not abiding by such rules, it was clear that individuals felt safeguarded from being blamed for taking the wrong decisions based on wrong judgements on behalf of entire households, compounds and communities under their responsibility.

One of the strengths of the programme was the community leaders' involvement in the sensitization and mobilization activities. However the approaches followed were not systematic or consistent, denying the potential to replicate the success stories and the opportunity to reflect on the pitfalls of the approach. Moreover, the community leaders' level of involvement was mostly dependant on their own will, regardless of the implementers' dedication or commitment, giving them a sense of powerlessness in dealing with refusals in the areas with weak or no community leaders' support.

The use of political structures without taking into account other influential entities within the communities, such as religious and traditional authorities, may pose a risk to the intervention's sustainability and transferability to other contexts where the population's political orientation is not as homogenous as that of Manhiça.

Some elements of the global and Southern African regional debates on options for malaria control strategies were identified at local level through this study. The most prominent discussion was regarding preferences between IRS and ITNs, which clearly pointed to the latter as the favourite intervention. However, the interests of the local communities have been weakly reflected on regional and national policy decision making processes, which continue to favour IRS over ITNs, as evidenced in a recent study on malaria vector policy in Southern Africa [36].

Additionally, environmental concerns and unwanted side-effects rising from DDT, also debated globally, did not seem to have reached the community-level debates, contrary to reports from other settings [17].

## Conclusion

The impact of IRS on malaria was weakly perceived at community level, as malaria control did not stand out among the perceived benefits of IRS. Moreover, the intervention's effectiveness was perceived as limited, and ITNs were preferred over IRS. The strongest factors associated with acceptability of IRS were the immediate reduction of insects, fulfilment of Governmental orientations, trust in the implementers, and community leaders' persuasion.

Despite the fact that the DDT debate has reached the global scale, this is not perceptible at local level, since the concerns regarding DDT and its toxicity were irrelevant in determining acceptability.

There is a need to reinforce messages relating IRS to malaria control, by confronting local expectations, misunderstandings and concerns, and to redefine the community involvement approaches, perhaps by borrowing from experiences with ITN distribution programmes, in order to make IRS a genuinely participative and sustainable programme from the community perspective.

## Competing interests

The authors declare that they have no competing interests.

## Authors' contributions

KM participated in the conception and design of the study, coordinated and participated in the data collection, performed the analysis, and drafted the manuscript. RP led the conception and design of the study, contributed to the analysis and interpretation, and revised the manuscript. CM participated in the design of the study, initiated the data collection, and participated in the analysis. CB oversaw and participated in the data collection, participated in the analysis. AN contributed to the design of the study, contributed to the manuscript. LF conducted data collection, participated in the analysis. CC oversaw and participated in the acquisition and analysis of the quantitative data, contributed to the manuscript. DN contributed to the manuscript. SM contributed to the manuscript. EM contributed to the manuscript. PA participated in the conception of the study, contributed in the interpretation of data, and critically revised the manuscript. All authors read and approved the final manuscript.
